# Germline activating MTOR mutation arising through gonadal mosaicism in two brothers with megalencephaly and neurodevelopmental abnormalities

**DOI:** 10.1186/s12881-015-0240-8

**Published:** 2015-11-05

**Authors:** Cameron Mroske, Kristen Rasmussen, Deepali N. Shinde, Robert Huether, Zoe Powis, Hsiao-Mei Lu, Ruth M. Baxter, Elizabeth McPherson, Sha Tang

**Affiliations:** Ambry Genetics Corporation, Aliso Viejo, CA 92656 USA; Marshfield Clinic Research Foundation, Marshfield, WI 54449 USA

**Keywords:** Diagnostic exome sequencing, Family-trio, MTOR, Autism, ASD, Macrocephaly, Megalencephaly, Gonadal mosaicism, Gain-of-function, Missense alteration

## Abstract

**Background:**

In humans, *Mammalian Target of Rapamycin (MTOR)* encodes a 300 kDa serine/ threonine protein kinase that is ubiquitously expressed, particularly at high levels in brain. MTOR functions as an integrator of multiple cellular processes, and in so doing either directly or indirectly regulates the phosphorylation of at least 800 proteins. While somatic MTOR mutations have been recognized in tumors for many years, and more recently in hemimegalencephaly, germline MTOR mutations have rarely been described.

**Case presentation:**

We report the successful application of family-trio Diagnostic Exome Sequencing (DES) to identify the underlying molecular etiology in two brothers with multiple neurological and developmental lesions, and for whom previous testing was non-diagnostic. The affected brothers, who were 6 and 23 years of age at the time of DES, presented symptoms including but not limited to mild Autism Spectrum Disorder (ASD), megalencephaly, gross motor skill delay, cryptorchidism and bilateral iris coloboma. Importantly, we determined that each affected brother harbored the MTOR missense alteration p.E1799K (c.5395G>A). This exact variant has been previously identified in multiple independent human somatic cancer samples and has been shown to result in increased MTOR activation. Further, recent independent reports describe two unrelated families in whom p.E1799K co-segregated with megalencephaly and intellectual disability (ID); in both cases, p.E1799K was shown to have originated due to germline mosaicism. In the case of the family reported herein, the absence of p.E1799K in genomic DNA extracted from the blood of either parent suggests that this alteration most likely arose due to gonadal mosaicism. Further, the p.E1799K variant exerts its effect by a gain-of-function (GOF), autosomal dominant mechanism.

**Conclusion:**

Herein, we describe the use of DES to uncover an activating MTOR missense alteration of gonadal mosaic origin that is likely to be the causative mutation in two brothers who present multiple neurological and developmental abnormalities. Our report brings the total number of families who harbor MTOR p.E1799K in association with megalencephaly and ID to three. In each case, evidence suggests that p.E1799K arose in the affected individuals due to gonadal mosaicism. Thus, MTOR p.E1799K can now be classified as a pathogenic GOF mutation that causes megalencephaly and cognitive impairment in humans.

**Electronic supplementary material:**

The online version of this article (doi:10.1186/s12881-015-0240-8) contains supplementary material, which is available to authorized users.

## Background

Autism spectrum disorders (ASDs) are a group of neuro-developmental maladies marked by social/communication impairments, repetitive behaviors, and sensory reactivity issues [1]. Even though the molecular etiology of autism is genetically heterogeneous, up to 10 % of cases are associated with a distinct genetic condition such as Fragile X syndrome, tuberous sclerosis, phenylketonuria, or Rett syndrome [2–5]. Furthermore, 20 % of autistic patients exhibit macrocephaly [6]. In a number of these cases, germline mutations in the tumor suppressor gene *PTEN* (*Phosphatase andTensin Homologue)* have been revealed to be the underlying cause of disease [7–11]. It has been estimated that the rate of *PTEN* disruption is up to 5 % in ASD patients with macrocephaly [12].

**Fig. 1:**
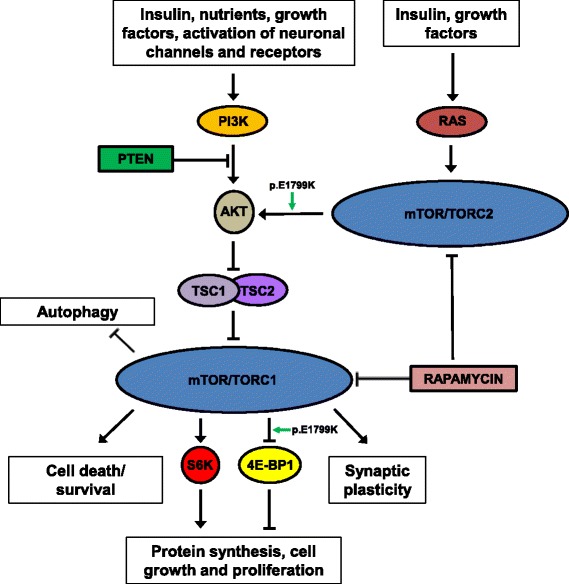
Simplified representation of the MTOR signaling network. Green arrows indicate the specific substrates shown to be phosphorylated/over-stimulated by p.E1799K-hyperactivated MTOR kinase. Other symbols and abbreviations used: stimulatory effects (); inhibitory effects (); 4E-BP1 (Eukaryotic Translation Initiation Factor 4E-Binding Protein 1); AKT (AKT/ Protein Kinase B); MTOR (Mammalian Target of Rapamycin); PI3K (Phosphoinositide 3-Kinase); PTEN (Phosphatase and Tensin Homolog); RAS (Rat Sarcoma Viral Oncogene Homolog); S6K (Ribosomal P70 S6 Kinase 1); TORC1 (MTOR Complex 1); TORC2 (MTOR Complex 2) TSC1 (Tuberous Sclerosis 1); TSC2 (Tuberous Sclerosis 2)

PTEN functions as a negative regulator of the PI3K (Phosphoinositide 3-Kinase)–AKT (AKT/Protein Kinase B)-MTOR (Mammalian Target of Rapamycin) signaling cascade, a simplified version of which is depicted in Fig. 1. At the center of this pathway lies MTOR, a 300 kDa serine/threonine protein kinase encoded by the *MTOR* gene, which in humans is situated on chromosome 1p36 [13]. By integrating upstream signals to downstream effector molecules like ribosomal P70 S6 (S6) and ribosomal P70 S6 kinase (S6K), the PI3K-AKT-MTOR axis serves to regulate a myriad of biological processes including cell growth and proliferation, apoptosis, protein synthesis, and transcription [14]. MTOR is known to mediate normal brain development by playing key roles in axonal and dendritic growth, and in the establishment and maintenance of synaptic plasticity [15].

As evidenced by the association of PTEN dysfunction with ASD and macrocephaly, hyper-activation of the PI3K-AKT-MTOR signaling cascade is an underlying factor for neurological disease and abnormal development. Such ‘TORopathies’ include neurological ailments like focal cortical malformations (FCM), tuberous sclerosis complex (TSC), and hemimegalencephaly, all of which are highly associated with epilepsy [16, 17]. Furthermore, hyperactivation of the MTOR axis has also been associated with somatic cancer [18].

Although specific molecular lesions have been defined for some TORopathies--for example, in cases where PTEN is disrupted--in many instances it remains difficult to pinpoint the exact molecular defect due to the complexity of the MTOR signaling network [17]. Even so, the emergence of next generation sequencing (NGS) has greatly facilitated the search for genes and pathogenic variants that underlie neurological disease. Several recent reports have detailed the successful use of whole exome sequencing (WES) to uncover both somatic and germline activating alterations within *MTOR* itself. [19–23] These clinical studies describe damaging *MTOR* alleles in unrelated probands who present overlapping neurological symptoms and brain defects [19–23]. In a recent cancer study, Grabiner and colleagues accessed publicly available sequencing data from tumor samples to uncover 33 *MTOR* alterations that conferred hyperactivation of the MTOR signaling pathway within cancer cells. These alterations clustered around six distinct regions in the C-terminal half of the protein [18].

Herein, we present the clinical and genetic investigation of two brothers affected with macrocephaly and ASD. Diagnostic Exome Sequencing (DES) identified a GOF MTOR alteration, p.E1799K, to be the causative mutation in this family. Co-segregation and short tandem repeat (STR) analyses revealed that MTOR p.E1799K likely originated as a consequence of gonadal mosaicism.

## Case presentation

### Clinical description

**Fig. 2:**
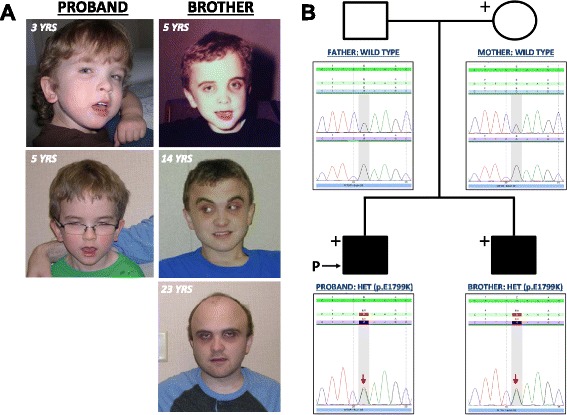
Clinical presentation and co-segregation analysis. **a**) Photographs of the proband (left column) and his affected brother (right column) at different ages. **b**) Familial co-segregation/ Sanger sequencing analyses indicate that the affected brothers are heterozygous for *MTOR* c.5395G>A, p.E1799K (red arrows); in contrast, both parents are homozygous for the wild type allele. These results strongly suggest that c.5395G>A (p.E1799K) arose due to gonadal mosaicism in one of the parents. Chromatograms are shown beneath each member of the pedigree. Note that ‘+’ indicates whole exome sequencing was performed, while open shapes represent unaffected family members; the black shapes represent the affected brothers

Table 1 lists the major clinical features displayed by the proband and his brother, as well as the features of additional patients who harbor MTOR p.E1799K but who are unrelated to the family described herein. The proband, who presented to medical genetics clinic at age 3 with macrocephaly, bilateral iris colobomas and gross motor delay, had a mildly unusual appearance, which was primarily due to macrocephaly with a prominent forehead (Fig. 2a, left panels). The proband was the product of an uncomplicated pregnancy and was large for his gestational age, with a weight of 2950 g (97 %) at 35 weeks; birth head circumference was not recorded. Since infancy, his linear growth and weight have been normal; however, macrocephaly was noted by 6 months, which prompted a head ultrasound. The result showed no evidence of hydrocephalus. Since 3 years of age, the proband’s head growth has been tracking parallel to, and 6 standard deviations above the mean. Investigation by magnetic resonance imaging revealed megalencephaly. Bilateral iris colobomas with normal posterior poles were noted at birth, and the proband wears glasses for strabismus. During infancy, he displayed hyperinsulinemia, which was managed through frequent feedings and administration of diazoxide. He continues to display asthma, as well as persistent food allergies that manifest through gastrointestinal and cutaneous symptoms.

**Table 1:** Clinical Findings Associated with MTOR E1799K^a^

	Proband	Sibling	Baynam 1	Baynam 2	Baynam 3
Lga^b^	+	+	-	+	+
Postnatal height/weight	Normal	Normal	Normal	Normal	Unknown
Macrocephaly	+5SD	+5SD	>+3SD	>+3SD	>+3SD
MRI	Megalencephaly	Megalencephaly	Mild ventricular prominence	Megalencephaly	Unknown
			Hypogenesis of corpus callosum	Perisylvian polymicrogyria,	
			Small pons & medulla	Hypogenesis of corpus callosum	
				Gray matter heterotopia	
Intellectual disability	Mild disability/autism	Moderate disability/autism	Hyperactive/speech delay	Marked global delay	Marked global delay
Seizures	-	-	+	+	+
Eye	Iris coloboma	Iris coloboma	-	-	-
Noonan-like face	-	-	+	+	+
Small chest/large abdomen	-	-	+	+	+

^a^This table does not include the case of an additional patient who harbors MTOR E1799K (Ghahramani *et al.*, ACMG 2015) due to lack of access to the patient's detailed clinical information

^b^Large for gestational age

The proband required surgery for undescended testes and an inguinal hernia. He did not display any unusual skin pigmentation in the genital area. Further, he has never displayed seizures. Initially hypotonic with delayed gross motor milestones, the proband walked at 18 months but remained somewhat hypotonic. His early language milestones appeared normal, but he subsequently exhibited significant social delays that were interpreted as mild ASD. Testing at age 7 revealed that his overall cognitive ability rated in the mildly impaired range. Previous genetic testing, which included PTEN sequencing and chromosomal microarray analysis, did not reveal any molecular abnormalities.

The proband’s older brother, who had previously displayed clinical features including macrocephaly, unilateral iris coloboma and autism, presented an unusual appearance, mainly due to severe macrocephaly, a prominent forehead, and deep set eyes surrounded by dark circles (Fig. 2a, right panels). The product of an uncomplicated pregnancy, the elder brother was born at 36 weeks and was large for his gestational age, with a weight of 3400 g (>97 %) and head circumference of 36 cm (>97 %). Although his subsequent linear growth and weight were normal, his macrocephaly nevertheless persisted. Since age 2, his head grew parallel to the norm but tracked 6 standard deviations above the mean, reaching an adult circumference of 65 cm. MRI scans revealed only megalencephaly. Although he is lactose intolerant and displays mild asthma, he does not display the severe allergies suffered by the proband. Unilateral iris coloboma was noted at birth and he has required several surgeries for strabismus; otherwise, his vision has been normal. In addition, he underwent surgery for undescended testes. The older brother has never had seizures; however, he was hypotonic and displayed significant delays in both speech and motor skills. Furthermore, repeated testing during childhood showed that he presented mild to moderate intellectual disability. His speech and social skills remained significantly below other developmental metrics, and as a teenager he was diagnosed with autism. Karyotype and Fragile–X test results were normal.

The brothers’ parents, who are healthy and non-consanguineous, possess normal intelligence, regular head circumference, and normal eyes. The father is of Norwegian, German and Bohemian descent, while the mother is of mixed European and Japanese ancestry. It should be noted that in addition to conceiving/giving birth to the proband and his brother, the parents/mother suffered a miscarriage due to trisomy 18. Furthermore, a maternal nephew displays mild autism despite having normal intelligence, and the paternal great grandmother was stated to have an iris coloboma, though no records are available to confirm this. No one else in the family has been reported to have macrocephaly or significant cognitive disability.

Due to the proband’s unique combination of symptoms, that is, macrocephaly and mild ASD, as well as normal *PTEN* sequencing results and normal comparative genomic hybridization (CGH) array results, DES was undertaken at age 6 years. We performed family trio-DES on genomic DNA (gDNA) isolated from the blood leukocytes of the proband, mother and brother. We then analyzed the resultant NGS data through bioinformatic filtering, inheritance modeling and finally, expert medical review. In so doing, we discovered that the likely molecular lesion underlying the brothers’ symptoms is the MTOR missense alteration, p.E1799K (c.5395G>A).

## Methods

### Statement of ethics

All research described in this case report was conducted in accordance with the World Medical Association Declaration of Helsinki. Furthermore, the clinical information presented herein was collected during the routine clinical care of a patient in the United States; thus, in accordance with US law, this study is exempt from Institutional Research Board approval. In order to perform the variant filtering and co-segregation analysis that are integral to the exome trio sequencing test, it is necessary to include relevant family member samples in the sequencing trio.  This format, which is currently a standard approach for clinical exome testing, is explicitly stated on the test requisition form. The mother of the patient approved the inclusion of all family members in the test. Further, all subjects provided signed, written informed consent to participate in this study. Written informed consent from the parents of the 6 year-old proband, as well as the written informed consent from the 23 year-old brother have been obtained for the publication of this case report and any accompanying images.

### Diagnostic exome sequencing (DES)

Genomic DNA was isolated from whole blood extracted from each family member utilizing the QIAsymphony gDNA extraction system (Qiagen, Hilden, Germany). Samples were prepared using either the SureSelect Target Enrichment System (Agilent Technologies, Santa Clara, CA) [24] or the SeqCap EZ VCRome 2.0 system (Roche NimbleGen, Madison, WI) [25]. Briefly, each DNA sample was sheared, blunt-end repaired, and ligated to indexed adapters. Utilizing solution-based hybridization with oligonucleotide probes, the coding exons and neighboring intronic sequence of each genome were enriched, while off-target sequences were washed away. The resultant enriched exome libraries were applied to the solid surface flow cell of an Illumina HiSeq 2000 sequencer (Illumina, San Diego, CA) for clonal amplification and sequencing using paired-end, 100-cycle chemistry.

### NGS data analysis

The acquisition and processing of data, alignment of sequence to Genome Reference Consortium Human genome build 37 (GRCh37), and bioinformatic filtering were performed as described in Farwell *et al.*, 2014 [26]. Initial data processing/base calling, including extraction of cluster intensities, was performed using RTA 1.12.4 (HiSeq Control Software 1.4.5). The sequence quality filtering script was executed using Illumina CASAVA software (ver 1.8.2, Illumina, Hayward, CA). Sequencing run metrics, including data yield (Mbases), %PF (pass-filter), number of reads, per cent of raw clusters per lane, and quality scores, were recorded in the Demultiplex_Stats.htm file and verified to surpass quality thresholds. Sequences were aligned to the human genome reference sequence GRCh37 and variant calls were generated using CASAVA and Pindel [27]. All exonic sequence and a minimum of 2 bases of flanking intronic sequence were analyzed. Data analysis focused on nonsense variants, small insertions and deletions, canonical splice site alterations, and non-synonymous missense alterations. Previously described gene mutations and polymorphisms were screened using resources that included the Human Gene Mutation Database Professional 2011.3 (HGMD 2011.3) [28], the Single Nucleotide Polymorphism database (dbSNP) [29], 1000 genomes [30], HapMap data [31] and NCBI resources such as PubMed. Importantly, the *in silico* filtering pipeline protected all variants annotated within the HGMD 2011.3 [28] and/ or the Online Mendelian Inheritance in Man (OMIM) database. Stepwise filtering included the removal of common Single Nucleotide Polymorphisms (SNPs), intergenic and 3′/5′ UTR (untranslated region) variants, non-splice-related intronic variants, and synonymous variants. Remaining variants were filtered for family history using models of inheritance. For every filtered variant, Ambry Variant Analyzer (AVA) was utilized to annotate the data, which included nucleotide and amino acid conservation; biochemical nature of the amino acid substitution; population frequency as tabulated in the ESP and 1000 genomes [30] databases; and finally, the impact of the nucleotide substitution on protein function, as predicted by the *in silico* modeling algorithms PolyPhen [32] and SIFT [33]. Importantly, each candidate alteration was reviewed by a molecular geneticist in order to assess the likelihood of pathogenicity. NGS alignments were viewed using the Integrative Genomics Viewer (IGV) software (Broad Institute, Cambridge, MA) [34].

### Microsatellite analysis

Short tandem repeat (STR) analysis was performed on each family member. Briefly, seven independent loci situated on separate chromosomes were amplified with fluorescently labeled primers and the resultant products electrophoresed on an ABI3730 DNA sequencer.

### Co-segregation analysis

Candidate alterations identified as potentially causative were confirmed using automated fluorescence dideoxy Sanger sequencing. Co-segregation analysis was performed on each available family member. Target specific primers were designed to include 5′ terminal sense and antisense sequencing tags. The gene-specific moieties of the primers were designed with the aid of PrimerZ [35]; oligonucleotides were synthesized by Integrated DNA Technologies (Coralville, IA) and sequencing was performed on an automated ABI3730 DNA sequencer (Life Technologies, Carlsbad, CA) according to standard procedures.

### Computational-structural modeling

The crystal structure of MTOR (PDB:4JSP) was used to model the location of the observed variant, MTOR p.E1799 [36]. Visualization of the local environment structure and overlap of the variant amino acid were performed in PyMOL (The Open Source PyMOL Molecular Graphics System, Version 1.7.x Schrödinger, LLC).

## Results and discussion

### Family trio DES coupled to bioinformatic analysis identifies MTOR p.E1799K as the likely genetic lesion

Exome sequencing of the family trio (proband, mother and brother) resulted in an average of 6.82 Gb being collected per sample. The average exome read depth/mean fold-coverage was 65.64 per sample, with an average of 93.13 % of bases covered at least 10-fold. On average, 81 % of bases had a quality score of Q30 or higher. Overall, the average base-call mean quality score for the family trio exceeded Q32 (Additional file 1: Table S1). Stepwise filtering of common SNPs, intergenic and 3′/5′ UTR variants, non-splice-related intronic variants, and synonymous variants resulted in approximately 11,000 variants being associated with each trio member (Additional file 2: Table S2). Subsequent filtering based on family history and inheritance modeling (autosomal dominant/recessive, X-linked dominant/recessive, and Y-linked) within the trio narrowed the list of candidates to 36 genes and 42 unique alterations (Additional file 3: Table S3). After manual review of each alteration in order to rule out sequencing artifacts and polymorphisms, followed by expert medical interpretation to rule out genes lacking clinical overlap, the list of candidates was narrowed to 2 genes, each with a unique alteration. These notable candidate genes, along with their respective alterations and corresponding *in silico* prediction scores, are presented in Table 2.

**Table 2:** Notable candidate genes/alterations

Gene	RefSeq Isoform	Variant/Coordinates^a^	SIFT Score (Prediction)	Polyphen2_HDIV Score (Prediction)	Polyphen2_HVAR Score (Prediction)
*Mammalian Target of Rapamycin (MTOR)*	NM_004958	Chr1:11190804C>T c.5395G>A p.E1799K	0.133 (Tolerated)	0.878 (Possibly Damaging)	0.463 (Possibly Damaging)
*Oligophrenin-1 (OPHN1)*	NM_002547	ChrX:67272394C>T c.2363G>A p.R788Q	0.130 (Tolerated)	0.995 (Probably Damaging)	0.457 (Possibly Damaging)

^a^Genomic coordinates correspond to GRCh37/hg19. Note that at the genomic level, both the *MTOR* and the *OLPHN1* variants are notated by their complementary bases. This is due to the fact that each gene is encoded by the minus strand of its respective chromosome

One of the candidates under consideration was an *Oligophrenin-1* (*OPHN1*) variant of uncertain significance (VUS), c.2363G>A (p.R788Q). Both the proband and his brother were hemizygous for this VUS, while their mother was heterozygous. *OPHN1* disruption has been shown to cause syndromic X-linked intellectual disability with cerebellar hypoplasia in affected individuals, and it also results in distinctive facial features [37]. Although one brother’s facial appearance demonstrated some similarity to the faces of *OPHN1-*mutated individuals, the overall set of traits displayed by the family herein is incongruous with the notion that the *OPHN1* VUS is the underlying cause of disease. For instance, both affected brothers display macrocephaly, which has not been previously attributed to mutations in *OPHN1*. Moreover, the lack of the key clinical finding of cerebellar hypoplasia in the affected brothers, as well as the absence of the usual carrier phenotype in the mother makes *OPHN1* an unlikely molecular candidate for disease. We therefore determined that the *MTOR* alteration c.5395G>A (p.E1799K), which is carried by both affected brothers but absent in the mother (Table 3), displayed sufficient clinical overlap with the proband’s phenotype to warrant further investigation/confirmation.

**Table 3:** Family trio DES identifies the MTOR alteration p.E1799K (c.5395G>A) in the proband and affected brother

Nucleotides^a^		Proband	Brother	Mother
Reference	C	53.23% (33/62)	52.63% (20/38)	100.00% (31/31)
Mutation	T	46.77% (29/62)	47.37% (18/38)	0.00% (0/31)
Q Score		220	148	119

^a^The variant is notated as C>T by the alignment software because the *MTOR* gene is situated on the minus strand of chromosome 1. For each trio member, the percentage of reads is followed in brackets by the fraction of wt or variant reads over the total number of reads

### Sanger sequencing confirms that p.E1799K co-segregates with disease in the family

As indicated by the red arrows shown in the lower two chromatograms of Fig. 2b, automated fluorescent dideoxy sequencing of gDNA extracted from family member blood samples confirmed the presence of the *MTOR* alteration c.5395G>A (p.E1799K) in the proband and his affected brother. In contrast, c.5395G>A (p.E1799K) was not detected in the peripheral blood mononuclear cell (PBMC) gDNA of either parent (Fig. 2b, upper chromatograms). Because the STR results are consistent with the reported familial relationships, these co-segregation data strongly suggest that the *MTOR* variant c.5395G>A (p.E1799K) originated as a consequence of gonadal mosaicism in one of the parents.

### MTOR dysfunction is concordant with the patients' phenotype

Overstimulation of the MTOR signaling network, as well as hyperactivation of MTOR itself have been shown to cause symptoms that overlap those displayed by the proband and his brother, namely macrocephaly and ASD [7–11, 23]. In humans, MTOR is expressed in every tissue of the body and is highly expressed in brain, where it plays important roles in axonal and dendritic growth, as well as in synaptic plasticity [15]. It is understood that the PI3K-AKT-MTOR signaling pathway plays a key role in mammalian growth and development via its ability to regulate protein translation (Fig. 1). Emerging evidence suggests that the MTOR axis regulates brain development by not simply acting as an ‘on/off’ switch that promotes beneficial protein synthesis, but rather by functioning as a valve that modulates translational rates during distinct temporal windows. Thus, if excessive protein synthesis occurs at the wrong time, it can result in significant deficits in synaptic plasticity and behavior [15].

Although there is ample evidence that other genes in the MTOR pathway, particularly *PTEN*, cause neurodevelopmental abnormalities including macrocephaly and autism, the first report of a pathogenic mutation in *MTOR* itself was a mosaic somatic mutation limited to the affected brain tissue of a child with hemimegalencephaly [19]. Subsequently, non-mosaic germline MTOR mutations were reported in two unrelated children with seizures and developmental delay, one of whom had documented megalencephaly and callosal dysgenesis [20, 21]. Recently, Baynam and colleagues reported three maternal half-siblings of Aboriginal Australian heritage who presented with megalencephaly, developmental delay, and epilepsy (Table 1). The affected individuals, who are unrelated to the family in this report, share the same germline activating MTOR alteration as that harbored by the affected brothers described herein: p.E1799K (c.5395G>A) [22]. The authors showed that the alteration was not present in the blood of the clinically normal mother and, as in the case of our proband and his affected brother, p.E1799K (c.5395G>A) was postulated to have arisen due to parental gonadal mosaicism [22]. Assays performed on stimulated PBMCs from one of the affected half-sisters revealed that MTOR activity was upregulated in her cells; moreover, this increase in activity could be inhibited/rescued back to normal levels through treatment with Rapamycin [22].

Emerging evidence indicates that altered MTOR activity may be associated with allergic reactions that are capable of triggering neuropathology. Because PTEN is an inhibitor of the PI3K-AKT-MTOR signaling cascade, loss-of-function (LOF) PTEN mutations result in increased MTOR activity, which in turn potentiates inflammation through ***1****)* microglial and mast cell proliferation [38, 39], and ***2****)* mast cell chemotaxis and activation [40, 41]. Theoharides *et al.* proposed a model in which children with hyperactivation of the MTOR pathway have a lower threshold for brain mast cell stimulation and are thus more sensitive to triggers like allergens, immune factors, neuro-hormones, stress and toxins [42]. In support of this model, the hormone Neurotensin (NT), which is secreted by the hypothalamus and functions as a stimulator of brain mast cells, is detected at increased levels in the serum of autistic children [43].

While the relationship between our patients’ colobomas and their MTOR mutation remains speculative, it is clear that the PI3K-AKT-MTOR pathway affects eye development. Most notably, Rieger anomaly is a cardinal feature of SHORT (Short Stature, Hyperextensibility, Hernia, Ocular Depression, Rieger Anomaly, and Teething Delay) syndrome, which is caused by heterozygous *Phosphatidylinositol 3-Kinase, Regulatory Subunit 1* (*PIK3R1*) mutations [44]. Cataracts are also an occasional feature of PTEN hamartoma syndrome [45].

### The MTOR variant p.E1799K is a GOF pathogenic mutation

The alteration p.E1799K, which was originally identified in multiple somatic tumor samples, has been empirically shown to cause hyperactivation of MTOR [18]. Interestingly, in HEK293 cells, p.E1799K selectively increases MTOR kinase activity towards the substrates AKT/ Protein Kinase B (AKT) and 4E-Binding Protein 1 (4E-BP1), but exerts minimal effect on MTOR kinase activity towards ribosomal P70 S6 Kinase 1 (S6K1) [18]. As illustrated in Fig. 1, increased phosphorylation of 4E-PB1 and AKT by TORC1 and TORC2, respectively, triggers increased protein synthesis. Moreover, constitutive or heightened MTOR activation has the potential to decouple and/or alter the manner in which the PI3K-AKT-MTOR signaling axis responds to neurotransmitters and external growth and development cues that exert their effects through neuronal cell surface receptor binding. Further support for the notion that increased MTOR activity is the mutational mechanism underlying neurological disease in the proband and his affected brother comes from the fact that homozygous LOF genotypes are tolerated in the healthy population [46].

**Fig. 3:**
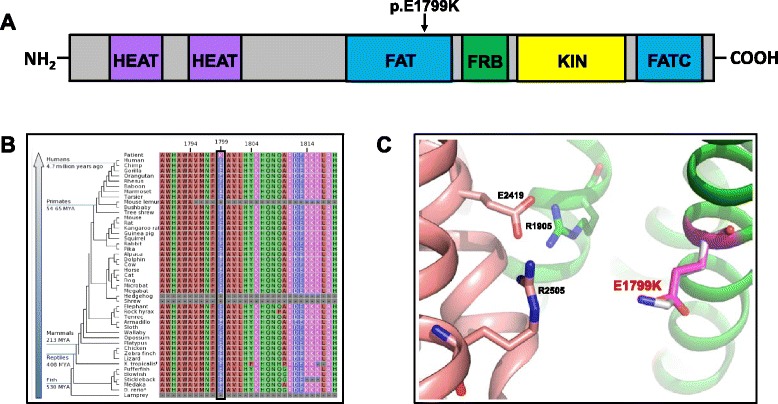
E1799K is a pathogenic activating mutation. **a**) Location of p.E1799K in relation to the domain/ structural organization of MTOR. Structural elements shown are the two groups of HEAT (Huntington Elongation Factor 3, A Subunit of PP2A TOR1) repeats; the FAT (FKBP12-Rapamycin-Associated Protein/TOR, Ataxia-Telangiectasia, Transactivation/ Transformation Domain-Associated Protein) domain; the FRB (FKBP12-Rapamycin Binding) domain; the KIN (Kinase) domain; and the terminal FATC (FAT C-Terminal) domain. **b**) The conservation plot reveals that E1799 (black rectangle) is evolutionarily conserved across vertebrates, which suggests that this amino acid plays an essential role for the normal function of MTOR kinase. **c**) A 3D schematic of the local environment surrounding the FAT-kinase domain interface that illustrates the mechanism by which p.E1799K causes hyperactivation of MTOR kinase. The FAT domain is represented in green while the kinase domain is peach-colored. Highlighted residues include E1799 (magenta stick), K1799 (white stick) and the activating residues described in the text: E2419, R1905 and R2505 (represented as sticks colored according to the domain in which they reside)

As shown in Fig. 3a, p.E1799K is situated in the FAT (FRAP, ATM, TRRAP) domain of MTOR. This domain adopts a c-shaped α solenoid structure that clamps onto the kinase domain (KD) of the enzyme and serves to negatively regulate MTOR activity [36]. Utilizing the MTOR crystal structure as a basis [36], we interpreted the mechanism by which p.E1799K leads to the hyperactivation of MTOR kinase. Glutamic acid 1799 (E1799), which is spatially positioned at the MTOR FAT-kinase domain interface, is directed towards Arginine 2505 (R2505), which is situated on the kinase side of this boundary (Fig. 3c). Due to their proximity, E1799 and R2505 likely interact through local electrostatic forces (<8 Å E1799 Oε2 – R2505 Nε), and this interaction contributes to the stringent repression of MTOR kinase activity by the FAT domain. We propose that the substitution of lysine for glutamic acid (Fig. 3c, white and magenta sticks, respectively), disrupts this electrostatic interaction and thereby destabilizes the local FAT-kinase environment to the point that the enzyme is shifted to a more active state. In fact, a mechanism similar to this has been shown to underlie the widely studied and structurally adjacent MTOR activating mutation, p.E2419K (Fig. 3c) [36, 47]. In this particular case, the substitution eliminates the E2419/R1905 salt bridge (not shown) that is normally present at the FAT-kinase interface. The loss of the salt bridge in turn reduces the negative regulation exerted by the FAT clamp on the KD, which results in increased kinase activity [36, 47]. Of therapeutic import is the fact that cancer derived cell lines and xenografts that carry the MTOR p.E1799K variant are hyper-responsive to Rapamycin treatment [18].

In support of the notion that MTOR p.E1799K is a pathogenic mutation that causes neurological disease in humans, Ghahramani and colleagues recently reported the same MTOR activating variant, p.E1799K, in an unrelated proband who displayed major clinical symptoms that were almost identical to those of the brothers’ in our report: macrocephaly and intellectual disability. Ghahramani’s group demonstrated that the p.E1799K mutation in their proband originated as a *de novo* germline event [23].

Additional evidence for the pathogenicity of MTOR p.E1799K comes from the fact that this variant is not present in the healthy human population. For instance, the following databases of human genetic variation make no reference of healthy individuals who harbor MTOR p.E1799K: the Exome Aggregation Consortium (ExAC), dbSNP, the 1000 Genomes database, and the Exome Sequencing Project (ESP) [29, 46, 48].

A number of independent clinical studies have documented patients who harbor causative MTOR missense variants and who present neurological and developmental symptoms that overlap those of the affected brothers in our study. For example, in a 2012 report, Lee *et al.* detailed a 5 year-old boy who suffered from seizures and whose brain displayed hemimegalencephaly (HME). Following lateral hemispherectomy, whole exome sequencing (WES) was performed on the patient’s excised brain tissue, as well as on his PBMCs. WES revealed that the proband harbored the somatic MTOR alteration p.C1483Y in his affected brain regions, but neither in his unaffected brain areas nor his PBMCs. Moreover, immunostaining revealed that the alteration in question resulted in hyperactivation of MTOR signaling. Since surgery, the patient has remained symptom-free. These results strongly suggest that the MTOR alteration p.C1483Y caused the patient’s symptoms [19]. Interestingly, a *de novo* germline MTOR alteration at the same position, p.C1483F, was identified in a 17-month old girl who displayed megalencephaly and intractable seizures [49]. These data are highly suggestive that cysteine 1483 plays an essential role for the maintenance of normal MTOR activity; moreover, substitution of p.C1483 for either isoleucine or phenylalanine potentiates the development of neurological disease in humans.

Other germline MTOR alterations have been reported in patients with epilepsy. For example, the *de novo* MTOR missense variant p.M1595I was detected in a non-verbal 18 month-old male proband who displayed infantile spasms, myoclonic seizures, and global developmental delay. Although no functional assays were undertaken in this case, multiple *in silico* models predicted the variant to be deleterious to normal protein function [20].

Because MTOR p.E1799K has been documented to be associated with overlapping neurological and developmental symptoms in independent families, and because it has been empirically demonstrated to cause hyperactivation of MTOR kinase, we propose that this variant is a pathogenic mutation that has the capacity to cause megalencephaly and neurological dysfunction in humans.

## Conclusions

Herein, we demonstrate that the MTOR missense variant p.E1799K is a pathogenic mutation that results in hyperactivation of MTOR kinase activity and causes megalencephaly/ macrocephaly and neurological impairment in humans. We demonstrate that p.E1799K exerts its effects on the developing brain by ***A****)* causing increased protein synthesis that is either exaggerated or decoupled from normal development cues during key temporal windows, and by ***B****)* potentially causing inflammation/ allergic reactions in the brain that could potentiate the development of focal lesions. It is of interest that both affected brothers in this study suffer from allergies. Finally, we suggest that because cells which carry the MTOR p.E1799K mutation are hyper-responsive to Rapamycin, treatment with this drug during post-natal development may prove beneficial in alleviating the craniofacial and neurological symptoms that develop in children who harbor hyper-activating MTOR mutations. Pre-clinical and clinical trials will be necessary to bear out this notion.

## Consent

The family described in this report has provided us with their written consent to have their clinical histories and photographs published.

## Additional files

Additional file 1: Table S1. Illumina Hiseq Run Metrics for family trio. (PPTX 75.6 kb) Additional file 2: Table S2. Bioinformatic Variant Filtering. (PPTX 54.9 kb) Additional file 3: Table S3. Variant Filtering Based on Inheritance Modeling & Interpretation. (PPTX 66.7 kb)

## Abbreviations

4E-BP1 AKT: Eukaryotic Translation Initiation Factor 4E-Binding Protein 1 AKT/ Protein Kinase B
ASD: Autism Spectrum Disorder
AVA: Ambry Variant Analyzer
CGH: Comparative Genomic Hybridization
dbSNP: Database of Single Nucleotide Polymorphism
DES: Diagnostic Exome Sequencing
ESP: Exome Sequencing Project
ExAC: Exome Aggregation Consortium
FAT: FKBP12-Rapamycin-Associated Protein/ TOR, Ataxia-Telangiectasia, Transactivation/ Transformation Domain-Associated Protein
FATC: FAT C-Terminal
FCM: Focal Cortical Malformation
FRB: FKBP12-Rapamycin Binding
gDNA: Genomic DNA
GOF: Gain-of-Function
GRCh37: Genome Reference Consortium Human Genome Build 37
HEAT: Huntington Elongation Factor 3, A Subunit of PP2A TOR1
HGMD 2011.3: Human Gene Mutation Database Professional 2011.3
ID: Intellectual Disability
KD: Kinase Domain
KIN: Kinase Domain
LOF: Loss-of-Function
MTOR: Mammalian Target of Rapamycin
NGS: Next Generation Sequencing
NT: Neurotensin
OMIM: Online Mendelian Inheritance in Man
OPHN1: Oligophrenin-1
PBMC: Peripheral Blood Mononuclear Cell
PI3K: Phosphoinositide 3-Kinase
PIK3R1: Phosphatidylinositol 3-Kinase, Regulatory Subunit 1
PTEN: Phosphatase and Tensin Homologue
RAS: Rat Sarcoma Viral Oncogene Homolog
S6: Ribosomal P70 S6
S6K: Ribosomal P70 S6 Kinase
SHORT: Short Stature, Hyperextensibility, Hernia, Ocular Depression, Rieger Anomaly, and Teething Delay
SNP: Single Nucleotide Polymorphism
STR: Short Tandem Repeat
TORC1: MTOR Complex 1
TORC2: MTOR Complex 2
TSC: Tuberous Sclerosis Complex
UTR: Untranslated Region
VUS: Variant of Uncertain Significance
WES: Whole Exome Sequencing
