# Supplementary figures and images for: Germline activating MTOR mutation arising through gonadal mosaicism in two brothers with megalencephaly and neurodevelopmental abnormalities
Source: BMC Med Genet. 2015 Nov 5;16:102. doi: 10.1186/s12881-015-0240-8 (PMC4635597; doi:10.1186/s12881-015-0240-8)

## Slide 1
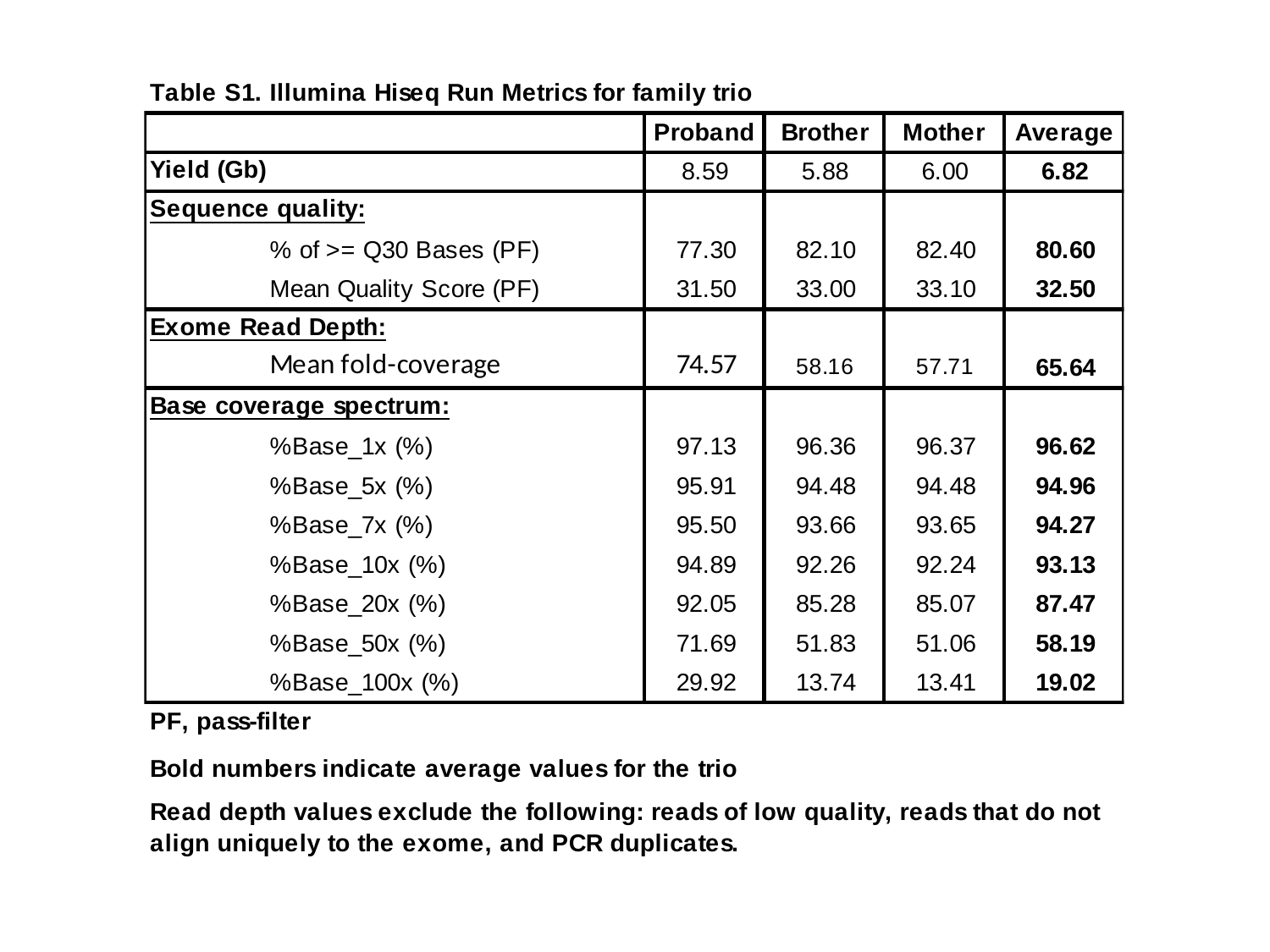

Supplement: Additional file 1: Table S1. — Illumina Hiseq Run Metrics for family trio. (PPTX 75.6 kb) [file 12881_2015_240_MOESM1_ESM.pptx]

## Slide 1
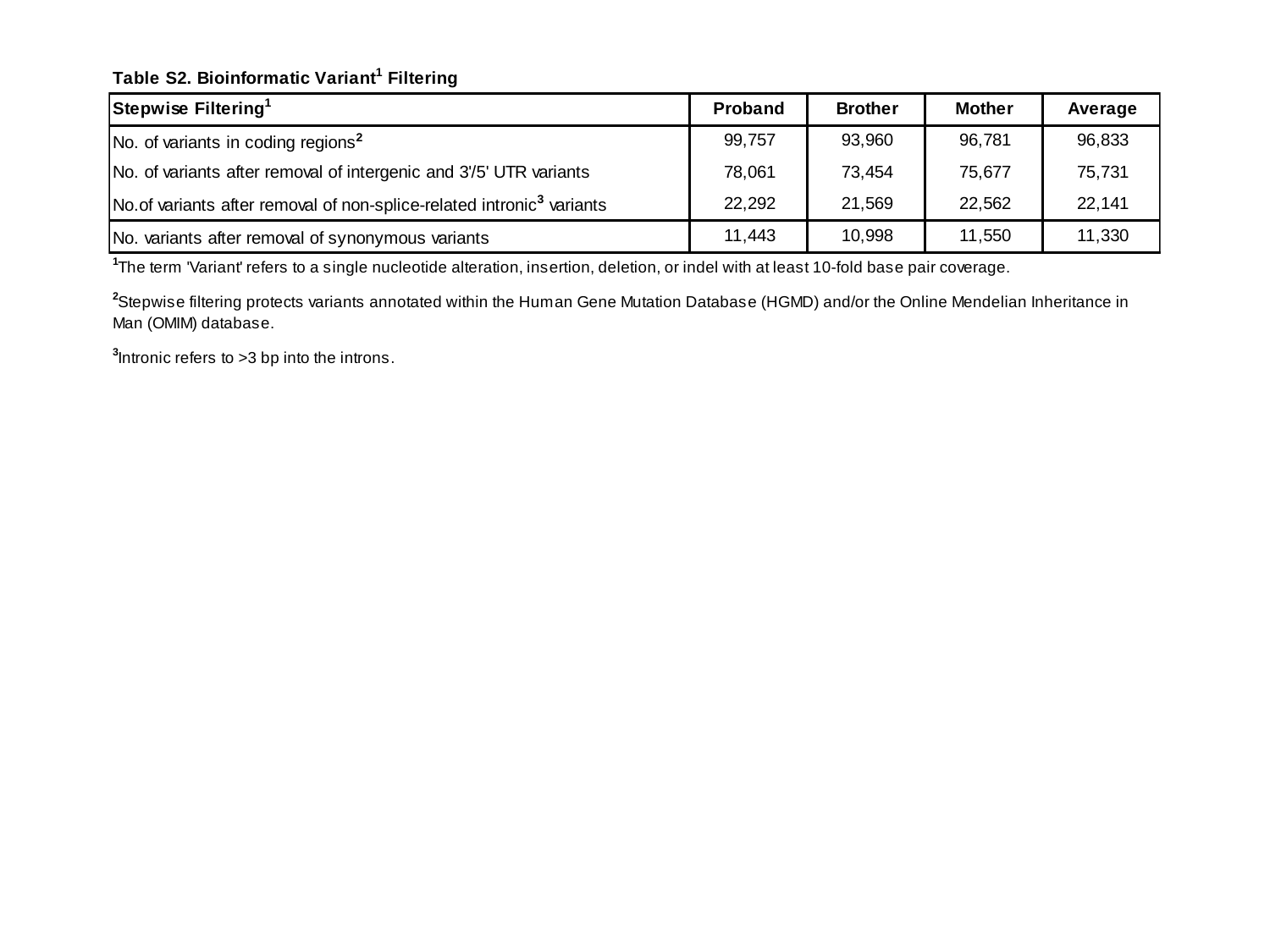

Supplement: Additional file 2: Table S2. — Bioinformatic Variant Filtering. (PPTX 54.9 kb) [file 12881_2015_240_MOESM2_ESM.pptx]

## Slide 1
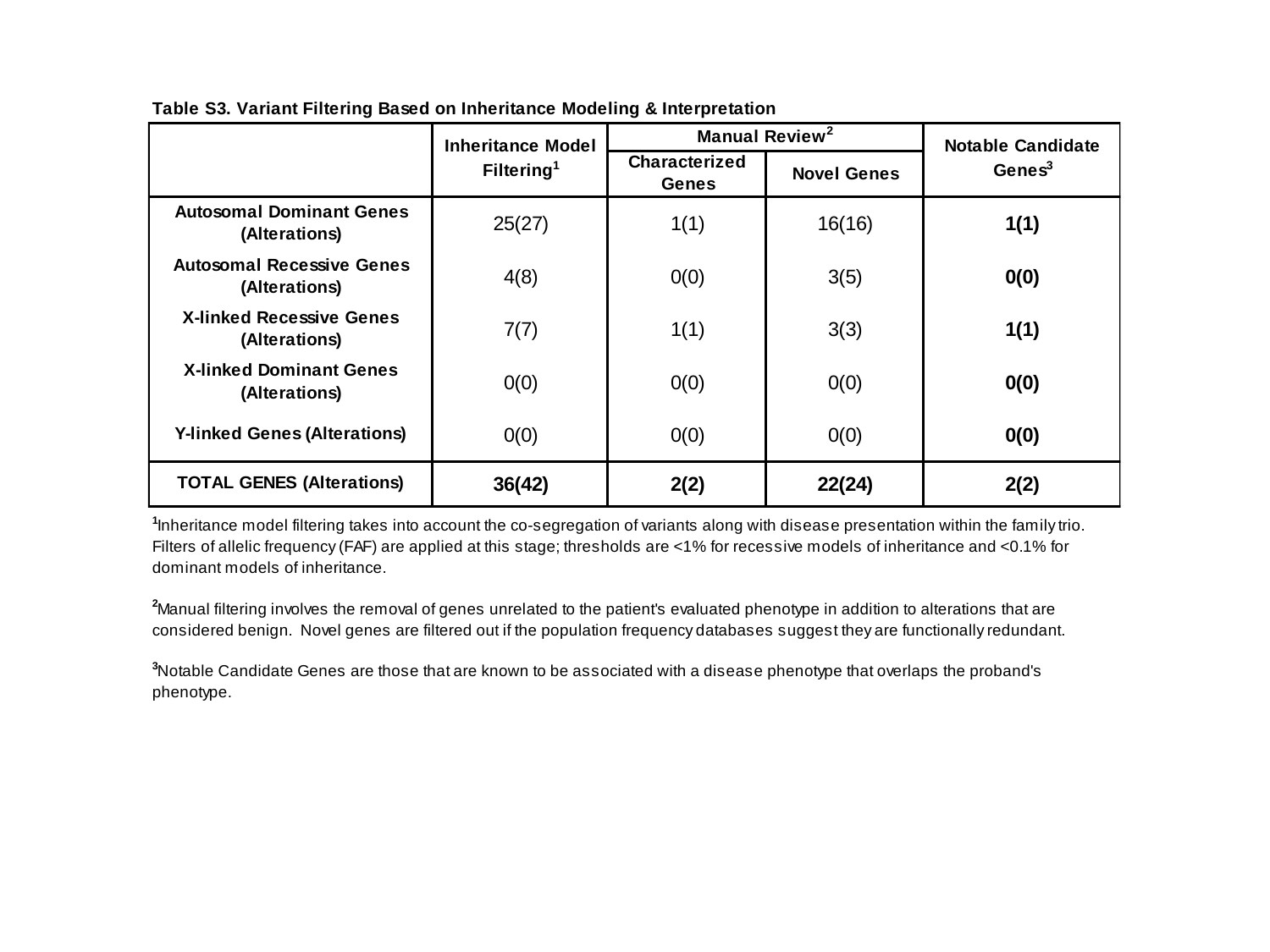

Supplement: Additional file 3: Table S3. — Variant Filtering Based on Inheritance Modeling & Interpretation. (PPTX 66.7 kb) [file 12881_2015_240_MOESM3_ESM.pptx]
